# Ultrasensitive Responses and Specificity in Cell Signaling

**DOI:** 10.1186/1752-0509-4-119

**Published:** 2010-08-25

**Authors:** Seth Haney, Lee Bardwell, Qing Nie

**Affiliations:** 1 Department of Mathematics, University of California at Irvine, Irvine, CA 92697, USA; 2 Department of Developmental and Cell Biology, University of California at Irvine, Irvine, CA 92697, USA; 3 Center for Complex Biological Systems, University of California at Irvine, Irvine, CA 92697, USA; 4 Center for Mathematical and Computational Biology, University of California at Irvine, Irvine, CA 92697, USA

## Abstract

**Background:**

Interconnected cell signaling pathways are able to efficiently and accurately transmit a multitude of different signals, despite an inherent potential for undesirable levels of cross-talk. To ensure that an appropriate response is produced, biological systems have evolved network-level mechanisms that insulate pathways from crosstalk and prevent 'leaking' or 'spillover' between pathways. Many signaling pathways have been shown to respond in an ultrasensitive (switch-like) fashion to graded input, and this behavior may influence specificity. The relationship of ultrasensitivity to signaling specificity has not been extensively explored.

**Results:**

We studied the behavior of simple mathematical models of signaling networks composed of two interconnected pathways that share an intermediate component, asking if the two pathways in the network could exhibit both *output specificity *(preferentially activate their own output) and *input fidelity *(preferentially respond to their own input). Previous results with weakly-activated pathways indicated that neither mutual specificity nor mutual fidelity were obtainable in the absence of an insulating mechanism, such as cross-pathway inhibition, combinatorial signaling or scaffolding/compartmentalization. Here we found that mutual specificity is obtainable for hyperbolic or ultrasensitive pathways, even in the absence of an insulating mechanism. However, mutual fidelity is impossible at steady-state, even if pathways are hyperbolic or ultrasensitive. Nevertheless, ultrasensitivity does provide advantages in attaining specificity and fidelity to networks that contain an insulating mechanism. For networks featuring cross-pathway inhibition or combinatorial signaling, ultrasensitive activation can increase specificity in a limited way, and can only be utilized by one of the two pathways. In contrast, for networks featuring scaffolding/compartmentalization, ultrasensitive activation of both pathways can dramatically improve network specificity.

**Conclusions:**

There are constraints to obtaining performance objectives associated with signaling specificity; such constraints may have influenced the evolution of signal transduction networks. Notably, input fidelity (preferential response to an authentic input) is a more difficult objective to achieve than output specificity (preferential targeting to an authentic output). Indeed, mutual fidelity is impossible in the absence of an insulating mechanism, even if pathways are ultrasensitive. Ultrasensitivity does, however, significantly enhance the performance of several insulating mechanisms. In particular, the ultrasensitive activation of both pathways can provide substantial improvement to networks containing scaffolding/compartmentalization.

## Background

To survive, and to function as a part of a whole organism, cells must sense and respond both to their environment and to other cells. Cells sense a variety of chemical and physical signals, that are then transmitted and interpreted in a signal-specific fashion. These signals include hormones such as insulin and adrenaline, growth factors such as EGF (epidermal growth factor) and NGF (nerve growth factor), and physical signals such as mechanical stress, osmotic pressure, light, pH, etc.

For particular signals to elicit appropriate responses (e.g. turn on certain genes), the news that a signal has been detected must be accurately relayed to the intracellular machinery necessary to evoke the response (e.g. transcription factors). Signal transmission (also called signal transduction) is generally initiated by the activation of a cell surface or intracellular receptor. The receptor then typically activates a cascade of intracellular kinases, which then regulate various downstream effectors. It is commonplace, however, for these intracellular kinases to be involved in more than one signaling cascade. The need to respond to a multitude of different signals, combined with a high promiscuity of kinases, creates a complicated and interconnected network of signaling components [1-4]. This interconnectedness leads to the potential for crosstalk and cross regulation, where the signals from one pathway intersect with another. Cross regulation can be beneficial or even necessary when cells must integrate their response to multiple signals simultaneously [5]. On the other hand, many signals necessitate a unique and decisive response, and a densely interconnected network may make such signal-exclusivity a difficult but vital objective to obtain [6-8]. Indeed, mutations that disrupt signaling specificity may play a role in the pathogenesis of cancer and other diseases [9,10].

Issues of signaling specificity feature prominently in mitogen-activated protein kinase (MAPK) cascade-mediated signaling [10-16]. A textbook example is the mammalian Ras/MAPK cascade, as exemplified using rat PC12 cells as a model system. This cascade is a central component of the response to both EGF and NGF; however, EGF causes the cells to proliferate, whereas NGF causes them to differentiate and sprout neuron-like projections [15,17-21]. Another textbook example is found in bakers/brewers yeast (*Saccharomyces cerevisiae*), where a set of overlapping MAPK cascades regulate mating, filamentous invasive growth, and stress-responses. In this case, elements of the same MAPK pathway are involved in transmitting at least three distinct signals: mating pheromone, nutrient limitation, and osmotic stress [13,21]. Even so, there is specificity from signal to cellular response: application of pheromone elicits mating (but not filamentation or stress response), nutrient limitation elicits filamentation (but not mating or stress response), and osmotic stress elicits only a stress response.

**Figure 1:**
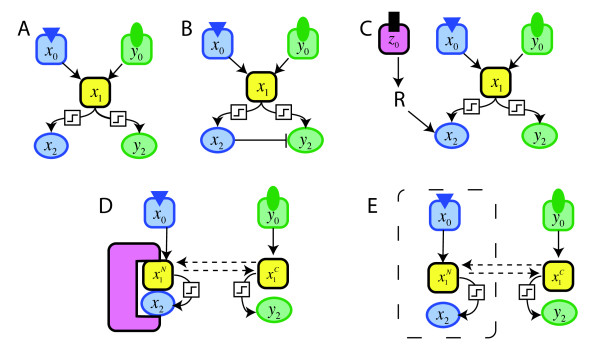
**Networks and insulating mechanisms**. **(A) **Schematic of a network with crosstalk. This network has no insulating mechanisms. However the connections between *x*_1 _and *x*_2 _and between *x*_1 _and *y*_2 _are allowed to be hyperbolic or ultrasensitive, denoted by . **(B-E) **Networks embellished with various insulating mechanisms **(B) **Cross Pathway Inhibition (CPI) from *x*_2 _to *y*_2_. **(C) **Combinatorial Signaling (CS) in the *X *pathway. **(D) **Scaffolding. **(E) **Compartmentalization.

How can the concept of signaling specificity be approached, modeled and quantified? Theoretical studies of signaling specificity have advanced our understanding in important ways. For instance, it was argued in [22] that the biological notion of 'signaling specificity' corresponds to two related yet distinct metrics: (1) the ability of pathways to preferentially activate their own output (*output specificity*); (2) the ability of pathways to preferentially respond to their own input (*input fidelity*). Within this framework, using the simplest possible architecture of an interconnected signaling network (denoted the "basic architecture, see Fig. 1A), and making the assumption that pathways are weakly-activated (which means that they can be modeled as linear systems [23,24]), it was shown that it is impossible for both pathways in the network to exhibit either input specificity or output fidelity [22,25].

How then do real signaling networks achieve specificity? Biochemical regulatory motifs knows as *insulating mechanisms *are thought to have evolved to maintain specificity by limiting 'leaking' or 'spillover' or 'bleed-through' between pathways. The fundamental insulating mechanism in cell regulation is specific protein-protein interactions [9,26,27], but this cannot account for specificity in networks containing pathways that share components. Insulating mechanisms that can buffer against spillover despite component sharing include combinatorial signaling, cross-pathway inhibition, compartmentalization, scaffolding, and kinetic insulation [22,25,28-30]. Some of the best experimental evidence for the existence and importance of these types of insulating mechanisms comes from the yeast MAPK network. In this system, multiple mutations that disrupt insulating mechanism function have been identified, and shown to result in increased levels of inappropriate signal crossover, often with adverse physiological consequences [29,31-37]. Theoretical and modeling studies have supported the idea that insulating mechanisms can provide varying degrees of output specificity and input fidelity to interconnected signaling networks [22,25,38-42].

**Figure 2:**
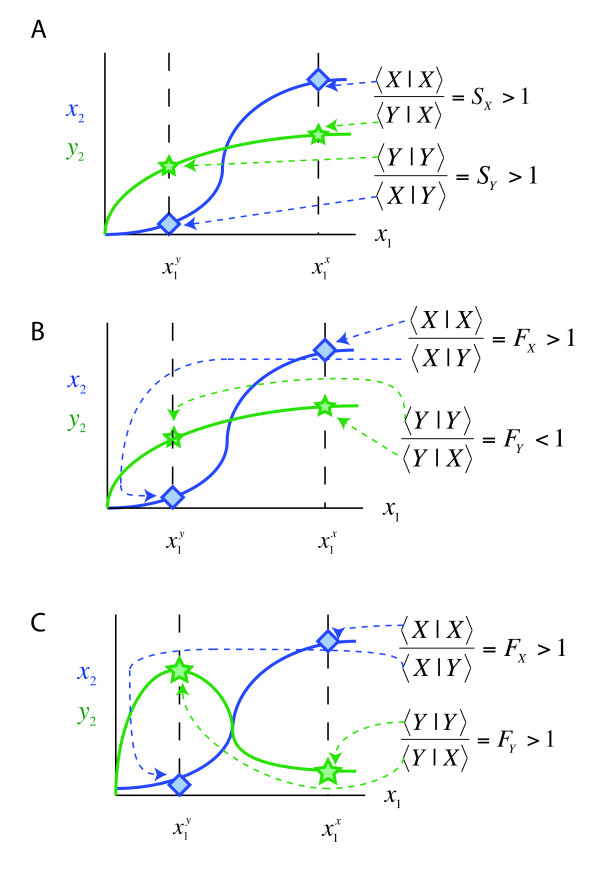
**Monotonic stimulus-response curves hinder mutual fidelity**. **(A) **Pictorial representation of a network employing only ultrasensitive activation achieving Mutual Specificity (MS). **(B) **Pictorial representation of a network without cross regulation that cannot attain Mutual Fidelity (MF). **(C) **A network with cross-pathway inhibition that attains MF.

Another performance objective often attributed to network-level properties of cell signaling pathways is the ability to respond to input in a switch-like manner [43-45]. This behavior is thought to endow certain pathways with the ability both to filter out input levels that are below some threshold value (such as might be caused by noise) and to respond dramatically to levels of input that have surpassed this threshold (see for example the solid blue line in Fig. 2A in comparison to the green line in the same figure). The term *ultrasensitive *refers to a situation where it takes a relatively small increase in input to cause a significant change in output [43,44]. This contrasts to *hyperbolic *or *Michaelian *input/output relationships, which require an 81-fold change in input to increase output from 10% to 90% maximal [43,44]. Hyperbolic relationships arise naturally from standard enzyme and binding kinetics, but mechanisms such as binding cooperativity and multisite phosphorylation can endow pathways with ultrasensitivity [45].

How do ultrasensitive (or hyperbolic) responses affect signaling specificity? Can ultrasensitivity provide signaling specificity to an interconnected network devoid of any insulating mechanisms? If not, does ultrasensitivity influence the performance of certain insulating mechanisms? These questions are challenging to address experimentally, so an approach using theory and modeling would seem a good starting point. Here we focus on simple phenomenological models of overlapping cascades that explicitly display various degrees of ultrasensitivity. The tractability of our approach allows us to derive analytic results that give a general insight into the effects of ultrasensitivity in achieving specificity. We find that ultrasensitivity cannot, by itself, provide specificity to an interconnected network, but that it can significantly enhance the performance of certain insulating mechanisms.

## Results and Discussion

### 1. Definitions of Mutual Specificity and Mutual Fidelity

As in previous treatments, [22,25], we consider a network consisting of two pathways, the *X *pathway and the *Y *pathway (Fig. 1A). Each pathway has a receptor/signaling component, *x*_0 _and *y*_0_, and a reporter/target component, *x*_2 _and *y*_2_. Notably, the two pathways share a common intermediate component, *x*_1_. Note that one component may be taken to represent the conglomeration of many chemical species. For example *x*_0 _may represent an entire G-protein-coupled receptor complex and several other steps upstream of a shared cascade *x*_1_. Hence, the network shown in Fig. 1A represents the simplest idealized "basic architecture" of a network in which two pathways share components. The input to each pathway will be given by specifying the levels of *x*_0 _and *y*_0_, and the output of each pathway will be measured by *x*_2 _and *y*_2_.

Let us denote the total output of pathway *X *when the cell is exposed to an × input signal (*x*_0 _> 0, *y*_0 _= 0) as *X*_out_|*X*_in_, read as '*X *output given *X *input', or simply '*X *given *X*'. In a similar fashion we define *Y_out_*|*Y_in_*, the value of *y*_2 _given that the *Y *pathway is activated (*x*_0 _= 0, *y*_0 _> 0). We also define the crosstalk terms *X_out_*|*Y_in _*(the value of *x*_2 _given that *Y *is activated) and *Y_out_*|*X_in_*. In this paper, we will use steady-state analysis so as to derive maximal analytical insight, so the outputs defined above refer to steady-state values.

These measures of output under different pathways inputs are used to express the metrics *specificity *and *fidelity *[22,25]. A pathway is said to have *output specificity *if that pathway's input activates its own output more than it does the output of any interconnected pathway. A pathway is said to have *input fidelity *if the output is greater when it receives its own signal than it is when it receives an interconnected pathway's signal. These two concepts can be quantified as:

(1.1) $$\begin{array}{cccc} S_{X}=\frac{X_{out}|X_{in}}{Y_{out}|X_{in}} & S_{Y}=\frac{Y_{out}|Y_{in}}{X_{out}|Y_{in}} & F_{X}=\frac{X_{out}|X_{in}}{X_{out}|Y_{in}} & F_{Y}=\frac{Y_{out}|Y_{in}}{Y_{out}|X_{in}} \end{array}$$

where *S*_*X *_denotes the output specificity in the *X *pathway and *F*_*Y *_denotes the input fidelity in the *Y *pathway, etc.

In order to escape obvious logical contradictions and function effectively, a signaling network needs to posses output specificity and input fidelity all its pathways simultaneously. To account for this in the context of a two-pathway network, we define three composite indicators, the degree of *Mutual Fidelity (MF)*, *Mutual Specificity (MS) *and *Mutual Fidelity & Mutual Specificity (MFMS):*

(1.2) $$\begin{array}{ccc} MS=\min\{S_{X},S_{Y}\} & MF=\min\{F_{X},F_{Y}\} & MFMS=\min\{MF,MS\} \end{array}$$

*MFMS *greater than 1 indicates that each of *S*_*X*_, *S*_*Y*_, *F*_*X *_and *F*_*Y *_are simultaneously (meaning they are evaluated using the same parameters including input levels and connection strengths) greater than 1, and hence the cell signaling network faithfully communicates through both pathways. Note that these definitions may be readily generalized to include more than two intersecting signaling pathways.

In the rest of this paper we explore methods utilized by biological systems to obtain *MFMS *greater than 1.

### 2. A Model with Ultrasensitivity

The scheme depicted in Fig. 1A can be modeled as a system of ordinary differential equations:

(2.1) $${\dot{x}}_{1}=a_{1}x_{0}+b_{1}y_{0}-d_{1}x_{1}$$

(2.2) $${\dot{x}}_{2}=a_{2}f^{X}(x_{1})-d_{2}^{x}x_{2}$$

(2.3) $${\dot{y}}_{2}=b_{2}f^{Y}(x_{1})-d_{2}^{y}y_{2}$$

These equations describe the formation of active signaling species *x*_1_, *x*_2 _and *y*_2_, and do not explicitly consider the inactive precursors from which they are converted. The parameters *a*_1 _and *a*_2 _are activation rate coefficients; *a*_2 _is proportional to the rate at which component *x*_1 _activates target *x*_2_. Similarly, $d_{1}^{x}$ and $d_{2}^{x}$ are deactivation (or decay) rate constants, and can be thought of as representing phosphatase activity or protein degradation, for example. The term ${\dot{x}}_{1}$ is a shorthand notation for$\frac{dx_{1}}{dt}$, the rate of change of component *x*_1 _at a particular moment in time. The functions *f*^*X *^and *f*^*Y *^are activation functions that describe how the rate of change with respect to time of *x*_2 _and *y*_2 _vary as a function of the concentration of active *x*_1_. For weakly-activated signaling pathways (i.e. pathways in which, at physiological levels of input, only a small fraction of any given component becomes activated), the production of *x*_2 _and *y*_2 _is a linear function of *x*_1_

$$f^{X}(x_{1})=f^{Y}(x_{1})=x_{1}$$

In contrast, for hyperbolic pathways, and for ultrasensitive pathways, the activation functions *f^X ^*and *f^Y ^*can often be reasonably approximated by Hill functions:

(2.4) $$\begin{array}{ll} f^{X}(x_{1})=\frac{{\left(x_{1}\right)}^{n}}{{\left(x_{1}\right)}^{n}+{\left(\varepsilon_{X}\right)}^{n}}\; & f^{Y}(x_{1})=\frac{{\left(x_{1}\right)}^{m}}{{\left(x_{1}\right)}^{m}+{\left(\varepsilon_{Y}\right)}^{m}} \end{array}$$

where the Hill exponents, *n *and *m*, quantify the degree of ultrasensitivity. For hyperbolic pathways, the Hill exponent is equal to 1, whereas for ultrasensitive pathways, the Hill exponent is greater than 1. Indeed, the greater the Hill exponent, the more switch-like the response. For a Hill number of 1, it an 81-fold change in input to increase output from 10% to 90% maximal. In contrast, for Hill numbers of 2 and 4, it takes a 9-fold and 3-fold change, respectively.

### 3. Hyperbolic or ultrasensitive signaling pathways can achieve Mutual Specificity

We will use the notation $x_{1}^{X}$ to refer to the steady state value of *x*_1 _given that pathway *X *is on (that is, activated) and *Y *is off; we could also have written *x*_1_|*X_in_*. Similarly, $x_{1}^{Y}$ refers to the steady state value of *x*_1 _when *X *is off and *Y *is on. As stated above, for weakly-activated pathways, activation kinetics are linear [23,24], and so $f^{X}(x_{1})=x_{1}^{X}$, and $f^{Y}(x_{1})=x_{1}^{Y}$. If we define the quantities $\alpha \equiv a_{2}/d_{2}^{x}$ and $\beta \equiv b_{2}/d_{2}^{y}$ (where α measures the connection strength from *x*_1 _to *x*_2_, and β from *x*_1 _to *y*_2_), we can then express the values of *S*_*X *_and *S*_*Y *_for the weakly-activated system simply as

$$S_{X}=(\alpha /\beta ),S_{Y}=(\beta /\alpha )$$

Hence, it is clear that any effort to increase *S*_*X *_will result in a reciprocal decrease of *S*_*Y*_, so that both *S*_*X *_and *S*_*Y *_cannot be simultaneously greater than one. Neither mutual specificity nor mutual fidelity is possible with the basic architecture and weak activation; thus, some sort of insulating mechanism is required to obtain *MFMS *for weakly-activated pathways [22].

When pathways are hyperbolic or ultrasensitive, mutual specificity becomes possible, even in the basic architecture. In these cases, the equations for *S*_*X *_and *S*_*Y *_are:

(3.1) $$\begin{array}{ll} S_{X}=\frac{\alpha }{\beta }\frac{{\left(x_{1}^{X}/\varepsilon_{X}\right)}^{n}+{\left(x_{1}^{X}/\varepsilon_{X}\right)}^{n}{\left(\varepsilon_{Y}/x_{1}^{X}\right)}^{m}}{{\left(x_{1}^{X}/\varepsilon_{X}\right)}^{n}+1}, & S_{Y}=\frac{\beta }{\alpha }\frac{{\left(x_{1}^{Y}/\varepsilon_{Y}\right)}^{m}+{\left(x_{1}^{Y}/\varepsilon_{Y}\right)}^{m}{\left(\varepsilon_{X}/x_{1}^{Y}\right)}^{n}}{{\left(x_{1}^{Y}/\varepsilon_{Y}\right)}^{m}+1} \end{array}\;$$

For hyperbolic but not ultrasensitive pathways, *n *= *m *= 1, and eqs. (3.1) reduce to

(3.2) $$\begin{array}{ll} S_{X}=\frac{\alpha }{\beta }\frac{\left(x_{1}^{X}/\varepsilon_{X})+(\varepsilon_{Y}/\varepsilon_{X}\right)}{(x_{1}^{X}/\varepsilon_{X})+1}, & S_{Y}=\frac{\beta }{\alpha }\frac{\left(x_{1}^{Y}/\varepsilon_{Y})+(\varepsilon_{X}/\varepsilon_{Y}\right)}{(x_{1}^{Y}/\varepsilon_{Y})+1} \end{array}$$

*S*_*X *_can be made large by setting α/β > > 1 and letting $(x_{1}^{X}/\varepsilon_{X})\to \infty$, whereas *S*_*Y *_can be made large by setting (ε*_X _*/ ε*_Y_*) > > 1 and letting $(x_{1}^{Y}/\varepsilon_{Y})\to 0$. In this case *S_X _*→ α/β and *S_Y _*→ (β · ε*_X_*)/(α · ε*_Y_*), which will both be greater than one so long as

$$1>(\alpha /\beta )>(\varepsilon_{x}/\varepsilon_{Y}).$$

With a careful selection of parameters mutual specificity of any degree can be obtained (see Additional file 1 section 1a). For ultrasensitive pathways, we have already seen that mutual specificity can be obtained, since hyperbolic pathways are a sub-case of ultrasensitive pathways. So, while both hyperbolic pathways and ultrasensitive pathways can achieve mutual specificity of any degree, ultrasensitive pathways impose less stringent requirements on parameters. For a detailed discussion of the advantages provided by ultrasensitivity see Additional file 1 section 1b.

A pictorial representation of a pathway with no cross-regulation obtaining mutual specificity is given in Fig. 2A. To reiterate, mutual specificity is possible in networks containing hyperbolic or ultrasensitive pathways, even when the topology of such networks is simply the basic architecture without any added insulating mechanism. However, as we show next, it is still impossible to attain mutual fidelity without adding some kind of insulating mechanism to the basic architecture.

### 4. Mutual Fidelity cannot be obtained by the basic architecture

If we assume that the activation functions *f^X ^*and *f^Y ^*are monotonic, but make no other assumptions as to their specific form, we can readily prove that mutual fidelity is impossible at steady state in the absence of an insulating mechanism. Let us consider the steady state of the system (see Fig. 2B and 2C for an illustration of the analysis below). Clearly *x*_1 _must take on different values given either *X *input or *Y *input, otherwise neither *X *nor *Y *fidelity would be possible at steady state. Suppose that $x_{1}^{X}>x_{1}^{Y}$. As the functions *f^X ^*and *f^Y ^*are activation functions, they are assumed to be monotonic and increasing, therefore more *x*_1 _gives more *x*_2 _and more *y*_2_. (We are assuming no other structure on the activation functions other than the fact that they are monotonic, therefore this result holds regardless of whether the functions are linear, Hill-like, or any other always-increasing function.). So if $x_{1}^{X}>x_{1}^{Y}$, then it must be that the steady state value of *x*_2 _given *X *input, *X_out_*|*X_in_*, must be greater than *x*_2 _given *Y *input, that is *X_out_*|*X_in _*>*X_out_*|*Y_in_*. This is, in fact, the definition of fidelity in the *X *pathway

(4.1) $$F_{X}=\frac{X_{out}|X_{in}}{X_{out}|Y_{in}}>1$$

Thus, fidelity in the *X *pathway is guaranteed. However, this same argument also implies that *Y_out_*|*X_in _*>*Y_out_*|*Y_in_*. This is exactly the statement that fidelity in the *Y *pathway

(4.2) $$F_{Y}=\frac{Y_{out}|Y_{in}}{Y_{out}|X_{in}}<1$$

is impossible.

It should be noted that the specification that this be evaluated at steady state is crucial to this conclusion. There are certain conceivable ways to utilize a time-dependent signal to allow for mutual fidelity and mutual specificity with certain types of activation functions without imposing added regulation.

Note that if we had instead assumed that $x_{1}^{X}>x_{1}^{Y}$, then we would have concluded that fidelity in the *Y *pathway is guaranteed, whereas fidelity in the *X *pathway is impossible. Therefore, we cannot have both *X *and *Y *fidelity, i.e. mutual fidelity, regardless of the form of the monotonic activation functions, *f^X ^*and *f^Y^*. In order to have mutual fidelity, one of the activation functions must be non-monotonic, that is, decreasing somewhere. This cannot be achieved by the basic architecture; it requires some type of added regulation.

### 5. Insulating mechanisms and cross-regulation

Biological signaling networks that share components are thought to contain one or more insulating mechanisms that provide specificity and fidelity [22,25,28-30]. From the analysis above it is clear that insulating mechanisms must be added if the basic architecture is to achieve mutual fidelity and mutual specificity. Here we briefly review three well-known insulating mechanisms, cross-pathway inhibition (CPI), combinatorial signaling (CS) and scaffolding/compartmentalization (SC) [22,25] (Fig. 1. We will then develop the notion of a cross-regulatory term that facilitates the comparison of different insulating mechanisms. Then, in subsequent sections, we address the effects of ultrasensitivity on the performance of these insulating mechanisms.

*Cross-pathway inhibition *occurs when one pathway inhibits another pathway. Here we consider a particular implementation of this, where both the inhibiting and inhibited components are downstream of a shared branchpoint (Fig. 1B). In the yeast MAPK network, both the MAP kinase Fus3 (an output specific to the mating pathway) and the transcription factor Tec1 (an output specific to the filamentation pathway) are downstream of the shared kinase cascade. Tec1 activation during mating is prevented, in part, because Fus3 phosphorylates Tec1 and thereby targets Tec1 for ubiquitin-mediated degradation [46-48]. Other likely examples of this type of cross-pathway inhibition in the yeast MAPK network include inhibition of Tec1 by the stress-response kinase Hog1 [49], and inhibition of Hog1 by the filamentation kinase Kss1 [37]. Following [22,25], we incorporate insulating mechanisms into the system composed of Eqs. (2.1)(2.2)(2.3). In cross-pathway inhibition, the equation for *y*2, (2.3), becomes

(5.1) $${\dot{y}}_{2}=b_{2}f^{Y}(x_{1})\left(\frac{1}{1+(x_{2}/\varepsilon_{g})}\right)-d_{2}^{y}y_{2}$$

Here production of *y*_2 _is inhibited by *x*_2_, with the amount of inhibition depending on the amount of *x*_2_. The parameter *ε*_*g *_is the IC50 (the inhibitory concentration 50%), which can be interpreted as the amount of *x*_2 _that results in 50% inhibition. When there is no *x*_2_, the production of *y*_2 _is unchanged; when *x*_2 _is much greater than *ε*_*g*_, *y*_2 _production is nearly completely shut off. Note that this insulating mechanism affects only the *Y *pathway's output and has no influence on *X *output. For a discussion of bi-directional mechanisms see Additional file 1 section 3.

In *combinatorial signaling*, in order for input from the *X *pathway to evoke a response, an independent input from a third receptor (*Z*) is required (see Fig. 1C). The component *x*_2 _acts a coincidence detector that only responds if both *x*_1 _and Z are active. In this case, the equation for *x*_2_, (2.2), becomes

(5.2) $${\dot{x}}_{2}=a_{2}R[x_{0}]f^{X}(x_{1})-d_{2}^{x}x_{2}$$

where

(5.3) $$R[x_{0}]\equiv \left\{\begin{array}{c} 1,\;if\;X\text{ is on }(\text{and }Y\text{ is off})\\ k_{leak},\;if\;Y\text{ is on},\;0\leq k_{leak}\leq 1 \end{array}\right\}.$$

Here, *R*[*x*_0_] represents the combinatorial input. As target *x*_2 _is a coincidence detector, its activity depends on two separate inputs, *R *and *x*_1_. If either input is zero, then *x*_2 _is also zero. When pathway *X *is on (and *Y *off), the coefficient R[*x*_0_] ≡ 1, and signal propagation through the network is identical to the basic architecture. When *Y *is on, *R*[*x*_0_] ≡ *k*_leak_, where *k*_leak_, a constant between zero and one, is the normalized basal level of signal flux from *Z*. Hence, *X_out_*|*Y_in _*will be reduced by a factor of *k*_leak _compared to the basic architecture. Hence, *k*_leak _= 1 has no specificity enhancing effect, whereas *k*_leak _= 0 completely eliminates *X *output given *Y *input. As with cross-pathway inhibition, combinatorial signaling only affects one output, in this case the *X *pathway output.

*Signaling scaffolds *are proteins that bind to two or more consecutively-acting components of a signaling cascade and, in so doing, facilitate signal transmission between them, (Fig. 1D). A prototypical example is the yeast Ste5 scaffold protein, which binds to all three tiers of the mating MAPK cascade [14]. We refer to this as the sequestering function of scaffolds, to be distinguished from the selective activation function of scaffold proteins [50], which resembles combinatorial signaling [25].

The sequestering function of scaffolds is implemented by expanding the system to include two different states of the "shared" component: bound to the scaffold (denoted $x_{1}^{N}$, for aNchored to the scaffold), and free in the cytosol (denoted $x_{1}^{C}$). It is presumed that active *x*_2 _can only be created by *x*_1 _that is bound to the scaffold, and that *x*_1 _bound to scaffold cannot create active *y*_2_. That is, *X *pathway output is a function of $x_{1}^{N}$ and the *Y *pathway output is a function of $x_{1}^{C}$, as shown below:

(5.4) $${\dot{x}}_{1}^{N}=a_{1}x_{0}-D_{\text{out}}x_{1}^{N}+D_{\text{in}}x_{1}^{C}-d_{1}^{x}x_{1}^{N}$$

(5.5) $${\dot{x}}_{1}^{C}=b_{1}y_{0}-D_{\text{in}}x_{1}^{C}+D_{\text{out}}x_{1}^{N}-d_{1}^{y}x_{1}^{C}$$

(5.6) $${\dot{x}}_{2}=f^{X}(x_{1}^{N})-d_{2}^{x}x_{2}$$

(5.7) $${\dot{y}}_{2}=f^{Y}(x_{1}^{C})-d_{2}^{y}y_{2}$$

The same set of equations can be used to describe the insulation mechanism of *compartmentalization *[22]. In compartmentalization, the *X *pathway is presumed to reside in one cellular compartment (e.g. the nucleus) and the *Y *pathway to reside in another (e.g. the cytosol). Leaking between the pathways can occur because the shared component can move between these two compartments to some extent. For instance, some portion of the pool of *x*_1 _activated in the nucleus ($x_{1}^{N}$) may move into the cytosol (becoming $x_{1}^{C}$), giving it the opportunity to inappropriately create *y*_2_. Thus, we refer to the insulating mechanism modeled by Eqns (5.4)-(5.7) as scaffolding/compartmentalization (SC). SC works by creating two different states for the shared component. These states are allowed to freely transform between one another:

$$X_{1}^{N}\overset{D_{out}}{\underset{D_{in}}{⇌}}X_{1}^{C}$$

SC becomes increasingly more effective as the exchange parameters *D_in_*, *D_out _*→ 0. At this limit, the *X *and *Y *pathways have no crosstalk, and hence possess perfect (i.e. infinite) *MFMS*.

#### Cross Regulatory Term (CRT)

In the following sections we will compare the effect of each of the above insulating mechanisms on the signaling pathway's ability to achieve *MFMS*, both numerically and analytically. In many cases one can show that arbitrarily high degrees of *MFMS *can be achieved at steady state. In other words for any *k *there is a set of parameters so that *MFMS *>*k*. However the realization of increasingly high degrees of *MFMS *requires more and more extreme choices of parameters and increasing cross-regulation. Therefore we need to be able to quantify the degree of additional regulation attributable to the insulating mechanism. Thus, for each of the different insulating mechanisms defined above, we identified a key dimensionless parameter to quantify the degree of additional regulation. We call this the Cross Regulatory Term (CRT); it is defined as follows:

(5.8) $$\begin{array}{l} \text{Cross-pathway inhibition }\left(\text{CPI}\right):\text{ CRT}\equiv \alpha /\varepsilon_{g}\\ \text{Combinatorial signaling }\left(\text{CS}\right):\text{ CRT}\equiv 1\;/k_{leak}\\ \text{Scaffolding/compartmentalization }\left(\text{SC}\right):\text{CRT}\equiv d_{1}/D \end{array}$$

where for SC we let *D_in _*= *D_out _*≡ *D *and $d_{1}^{x}=d_{1}^{y}\equiv d_{1}$.

Each of the CRTs were chosen intuitively as a set of parameters that quantifies the cross pathway regulation. For example with combinatorial signaling, the leak rate is clearly the parameter that quantifies the cross pathway regulation, as it is the only parameter that differentiates a CS network from the basic architecture. Both numerical (data not shown) and analytic results (see below) show that the CRTs as defined are in fact critical for determining specificity.

### 6. Ultrasensitivity can improve insulating mechanism performance

As we have seen, mutual fidelity at steady state is impossible without some kind of additional regulation. In this section we derive maximal values for *MFMS *for each of the insulating mechanisms, for networks with both linear and ultrasensitive activation. As stated above, in many cases one can show that arbitrarily high degrees of *MFMS *can be achieved at steady state. Here we derive bounds based on a fixed CRT. We also numerically evaluate the steady state values for each network at different levels of CRT to show that the bounds we derive are in fact sharp.

#### Linear Activation

For linear activation, deriving expressions for each of the specificity indicators has been done previously [25]. Here we shall re-formulate these in terms of the CRT.

For cross-pathway inhibition, mutual fidelity is not possible; in other words, *Y *fidelity implies that there is no *X *fidelity, and vice versa.

$$\begin{array}{ll} F_{X}=\frac{x_{1}^{X}}{x_{1}^{Y}}, & \;F_{Y}=\frac{x_{1}^{Y}/x_{1}^{X}+\left(CRT\right)x_{1}^{Y}}{1+\left(CRT\right)x_{1}^{Y}} \end{array}$$

Upon inspection *F_Y _*> 1 only when $x_{1}^{Y}>x_{1}^{X}$, which then makes *F_X _*< 1. Therefore, regardless of the CRT, *MFMS *≤ 1.

In the case of combinatorial signaling, however, one can show that $MFMS\leq \sqrt{CRT}$. In this case

$$MFMS=\min\left\{\left(\frac{\alpha }{\beta }\right),\;\left(\frac{\beta }{\alpha }\right)CRT,\;\left(\frac{x_{1}^{X}}{x_{1}^{Y}}\right)CRT,\left(\frac{x_{1}^{Y}}{x_{1}^{X}}\right)\right\}.$$

The maximum of this expression over all of the parameters occurs when $\alpha /\beta =x_{1}^{Y}/x_{1}^{X}=\sqrt{CRT}$, and at this point $MFMS=\sqrt{CRT}$.

For scaffolding/compartmentalization (SC), the output specificity and input fidelity readily are calculated:

$$\begin{array}{llll} S_{X}=\left(\frac{\alpha }{\beta }\right)\frac{x_{1}^{N}|X_{in}}{x_{1}^{C}|X_{in}}, & S_{Y}=\left(\frac{\beta }{\alpha }\right)\frac{x_{1}^{C}|Y_{in}}{x_{1}^{N}|Y_{in}}, & F_{X}=\frac{x_{1}^{N}|X_{in}}{x_{1}^{N}|Y_{in}}, & F_{Y}=\frac{x_{1}^{C}|Y_{in}}{x_{1}^{C}|X_{in}}. \end{array}$$

Evaluating these expressions, we find:

$$\begin{array}{c} \frac{x_{1}^{N}|X_{in}}{x_{1}^{C}|X_{in}}=\frac{x_{1}^{C}|Y_{in}}{x_{1}^{N}|Y_{in}}=1+\frac{d_{1}}{D}=1+CRT,\\ \begin{array}{ll} \frac{x_{1}^{N}|X_{in}}{x_{1}^{N}|Y_{in}}=\frac{a_{1}x_{0}}{b_{1}y_{0}}(1+CRT), & \frac{x_{1}^{C}|Y_{in}}{x_{1}^{C}|X_{in}}=\frac{b_{1}y_{0}}{a_{1}x_{0}}(1+CRT) \end{array} \end{array}$$

Hence we obtain *MFMS *≤ 1 + *CRT*.

**Figure 3:**
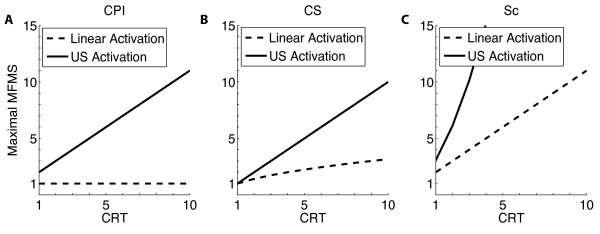
**Maximal values for mutual fidelity & mutual specificity (MFMS)**. Maximal values for MFMS under cross-pathway inhibition (CPI, **A)**, combinatorial signaling (CS, **B**) and scaffolding/compartmentalization (SC, **C**) are graphed as a function of the cross-regulatory term (CRT). Networks with linear activation are graphed in dashed lines while networks with ultrasensitive (US) activation are graphed in solid lines. The bound for the network with ultrasensitive activation and SC is plotted using the formula (6.4) with *a *= 2 and *n *= 2. More dramatic results occur with higher values of *n*.

These bounds, (Fig. 3 dashed lines), are sharp, or the most accurate upper bound, as is apparent from how they are derived. Below we show that the bounds derived for networks with ultrasensitive activation greatly supercede these values.

#### Ultrasensitivity

In the case of ultrasensitive activation for cross-pathway inhibition (CPI), we can obtain a simple bound on *MFMS*. Due to the fact that *MFMS *is the minimum of four quantities the maximum of *MFMS *is at most as big as the smallest of *S_X_*, *S_Y_*, *F_X _*and *F_Y_*. In the case of CPI it is easiest to bound *MFMS *by *F_Y _*(In the Additional file 1 we show that this bound for *MFMS *is sharp: in that there is a choice of parameters so that the *MFMS *is arbitrarily close to it. See section 2 in the Additional file 1 for derivation).

$$F_{Y}=\frac{Y|Y}{Y|X}=\frac{f^{Y}(x_{1}^{Y})}{f^{Y}(x_{1}^{X})}\frac{\left(1+CRT\cdot f^{X}(x_{1}^{X})\right)}{\left(1+CRT\cdot f^{X}(x_{1}^{Y})\right)}<\frac{\left(1+CRT\cdot f^{X}(x_{1}^{X})\right)}{\left(1+CRT\cdot f^{X}(x_{1}^{Y})\right)}<1+CRT\cdot f^{X}(x_{1}^{X})<1+CRT$$

Therefore we can assert

(6.1) MFMS<1+CRT.

Hence, in contrast to the case with weak-activation and cross-pathway inhibition, where mutual fidelity was impossible, when ultrasensitive, or even hyperbolic, activation is added to this architecture, *MFMS *> 1 can be obtained.

For combinatorial signaling (CS), the case is much simpler. Regardless of the parameter choice either $x_{1}^{X}>x_{1}^{Y}$ and therefore $f^{Y}(x_{1}^{X})>f^{Y}(x_{1}^{Y})$ and thus

$$F_{Y}=\frac{f^{Y}(x_{1}^{Y})}{f^{Y}(x_{1}^{X})}<1,$$

or $x_{1}^{Y}>x_{1}^{X}$ and therefore $f^{X}(x_{1}^{Y})>f^{X}(x_{1}^{X})$ and thus

$$F_{X}=\frac{f^{X}(x_{1}^{X})}{f^{X}(x_{1}^{Y})}CRT<CRT.$$

Thus in any case we have,

(6.2) MFMS<CRT

In both of these cases the degree to which ultrasensitivity helps is hidden. While the bounds for the hyperbolic(*n *= *m *= 1) and ultrasensitive case are the same, the speed at which they approach these bounds is much different. With high Hill exponents the constraints on the remaining parameters are much less stringent (see Additional file 1 section 1b). Further high degrees of ultrasensitivity can drastically decrease one of the crossterms *X_out_*|*Y_in _*or *Y_out_*|*X_in_*, see more on this in the next section.

For scaffolding/compartmentalization (SC), making a similar type of bound is less fruitful. Fortunately the exact formula for each of the specificity indicators can be derived straightforwardly. In the case of a symmetric parameter choice, where we let many of the parameters from the *X *pathway be the same as those in the *Y *pathway, i.e.

(6.3) $$\begin{array}{llll} a_{1}=b_{1}\equiv a, & \alpha =\beta , & n=m, & \varepsilon_{X}=\varepsilon_{Y}=1 \end{array},$$

we obtain the bound

(6.4) $$MFMS=\frac{a^{n}{\left(1+CRT\right)}^{n}+{\left(1+CRT\right)}^{n}{\left(2+CRT\right)}^{n}}{a^{n}{\left(1+CRT\right)}^{n}+{\left(2+CRT\right)}^{n}}∼{\left(1+CRT\right)}^{n}.$$

In this case, unlike the cases of CPI or CS, the ultrasensitivity and CRT contributions to *MFMS *are intimately connected. This connection creates a super-linear increase in *MFMS *due to increasing CRT when ultrasensitivity is greater than one, in contrast with both CPI and CS where *MFMS *increases only linearly in CRT regardless of the degree of ultrasensitivity. This means that for networks with scaffolding/compartmentalization, even with a low value of the CRT, sufficient ultrasensitivity can serve to greatly increase *MFMS*, and visa versa. Note that in this symmetric case we have not derived a bound, the *MFMS *is in fact equal to this value. This is because the symmetric parameter choice greatly simplifies the situation by making *S*_*X *_= *S*_*Y *_= *F*_*X *_= *F*_*Y *_= *MFMS*.

Numerical evaluation of the specificity indicators confirm the bounds derived for the networks with ultrasensitive activation are also sharp (data not shown). Further numerical simulation shows that, in the case in which only symmetric parameters are used, as in (6.3), both the maxima and distribution of *MFMS *values are similar; so the results derived in this case should be representative of the more general case.

The bounds are plotted in Fig. 3 for comparison with those with linear activation. In each case the bounds with ultrasensitive activation clearly supercede those with linear activation. In particular, note the steep increase in *MFMS *due to the super-linear dependence on CRT in the case of scaffolding/compartmentalization.

**Figure 4:**
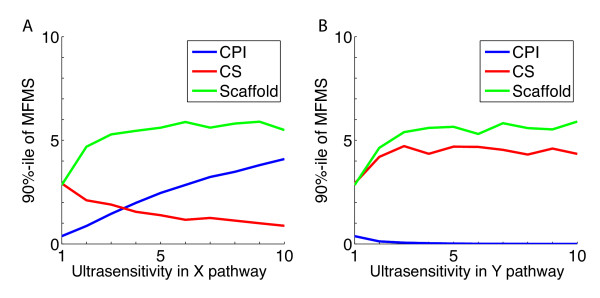
**Effect of increasing ultrasensitivity on mutual fidelity & mutual specificity**. *MFMS *values for networks with CPI, CS, and SC were calculated for 10,000 trials over a large range of parameters (See below for parameter ranges). *MFMS *values at the 90^th ^percentile of the distribution were then plotted as a function of degree of various Hill exponents. **(A) **Dependence of each type of network on *n*, the degree of ultrasensitivity in the *X *pathway, where ultrasensitivity in the *Y *pathway was set to one, *m = 1*. **(B) **Dependence on *m*, the ultrasensitivity of the Y pathway, where *n = 1*. **(Parameter Ranges)**: $\begin{array}{l} CPI:\left\{\begin{array}{ccccc} 1\leq a_{1}/d_{1}\leq 5, & 0.1\leq b_{1}/d_{1}\leq 1, & 0.1\leq a_{2}/d_{2}^{X}\leq 5, & 0.1\leq b_{2}/d_{2}^{Y}\leq 5, & .01\leq \varepsilon_{Y}\leq 10 \end{array}\right\},\\ CS:\left\{\begin{array}{ccccc} 0.1\leq a_{1}/d_{1}\leq 1, & 1\leq b_{1}/d_{1}\leq 5, & 0.1\leq a_{2}/d_{2}^{X}\leq 5, & 0.1\leq b_{2}/d_{2}^{Y}\leq 5, & .01\leq \varepsilon_{Y}\leq 10 \end{array}\right\},\\ Sc:\left\{\begin{array}{ccccc} 1\leq a_{1}/d_{1}\leq 5, & 1\leq b_{1}/d_{1}\leq 5, & 0.1\leq a_{2}/d_{2}^{X}\leq 5, & 0.1\leq b_{2}/d_{2}^{Y}\leq 5, & .01\leq \varepsilon_{Y}\leq 10 \end{array}\right\}. \end{array}$.

To investigate the case where the degree of ultrasensitivity is the limiting factor on *MFMS*, we numerically evaluated the effect of independently increasing the *n *and *m *exponents, while holding the CRT constant and sufficiently high. For each of the insulating mechanisms, *MFMS *was calculated numerically over a large range of parameters and basic statistics were used. As shown in Fig. 4A and 4B, only *n *increases *MFMS *in the case of CPI and only *m *does this for CS, just as derived in the above bounds. In contrast, for scaffolding/compartmentalization, increasing either *n *or *m *increased *MFMS*. In the case where only one of the Hill exponents is large while the other is kept small the SC network does no better than CPI or CS (data not shown). This is due to the fact that *MFMS *is a minimum of the four specificity indicators, (1.2), and thus is constrained by the smallest one. However if both *n *and *m *are increased simultaneously, *MFMS *for scaffolding/compartmentalization increases rapidly.

In the case with scaffolding, high degrees of *MFMS *are achieved at relatively low levels of ultrasensitivity, *n *or *m*. For scaffolding with *n *= *m *= 10, *MFMS *is well over 100 (data not shown).

### 7. Strategies to maximize MFMS using ultrasensitivity

In this section we wish to understand why increasing only one Hill exponent is beneficial for CPI and CS, whereas increasing both is beneficial for scaffolding/compartmentalization as shown in section 6. To explain these observations we study how responsive the specificity and fidelity indicators are to changes in *n *and *m *by analyzing their partial derivatives.

If ultrasensitivity is helpful to *MFMS*, then increasing *n *and *m *should increase *S*_*X*_, *S*_*Y*_, *F*_*X *_and *F*_*Y*_. (See section 4 of Additional file 1 for derivation. This same approach can be taken on the steady states of *x*_2 _and *y*_2 _directly with the same result we derive below). First observe the result in the case where there are no insulating mechanisms. Taking derivatives:

(7.1) $$\begin{array}{ll} \frac{\partial S_{X}}{\partial n}>0\Leftrightarrow x_{1}^{X}>\varepsilon_{X} & \frac{\partial S_{Y}}{\partial n}>0\Leftrightarrow x_{1}^{Y}<\varepsilon_{X}\\ \frac{\partial S_{X}}{\partial m}>0\Leftrightarrow x_{1}^{X}<\varepsilon_{Y} & \frac{\partial S_{Y}}{\partial m}>0\Leftrightarrow x_{1}^{Y}>\varepsilon_{Y} \end{array}$$

Combining these conditions gives,

(7.2) $$x_{1}^{X}<\varepsilon_{Y}<x_{1}^{Y}<\varepsilon_{X}<x_{1}^{X}$$

which is a necessary and sufficient condition for both *n *and *m *to have positive effects on both *S*_*X *_and *S*_*Y*_. When this technique is applied to the fidelity indicators we derive the same conditions. Clearly, not all these conditions can be satisfied simultaneously, since either $x_{1}^{X}>x_{1}^{Y}$ or $x_{1}^{X}>x_{1}^{Y}$.

After the addition of insulating mechanisms the exact derivatives change slightly, but Eqn. (7.2) still holds for both cross-pathway inhibition (CPI) and combinatorial signaling (CS). In other words adding either of these insulating mechanisms, or both simultaneously (see Additional file 1 section 3) will not change the fact that the equations in (7.1) cannot be simultaneously satisfied.

In CPI, both *Y_out_*|*X_in _*and *Y_out_*|*Y_in _*are decreased due to the inhibition by *x*_2_, which will allow for *S_X _*> 1 and potentially *F_Y _*> 1, but CPI does nothing to decrease the *X_out_*|*Y_in _*term. Thus in order to obtain fidelity in the × pathway, *F_X _*> 1, and hence *MFMS *> 1 it must be the case that:$\varepsilon_{y}<x_{1}^{y}<\varepsilon_{x}<x_{1}^{x}$. Under this parameter choice, increasing *n *has only favorable effects, but increasing *m *has mixed effects; it increases *S_Y _*but decreases *S_X_*. In the case of CS the relation is the opposite because CS only effects the *X_out_*|*Y_in _*term and has no effect on the others. So the parameters must satisfy the relations: $\varepsilon_{x}<x_{1}^{x}<\varepsilon_{y}<x_{1}^{y}$. Again if this were not the case we would not attain *MFMS *> 1, this time because *F_Y _*< 1. The consequence of this parameter choice, however, is that increasing *n *decreases *S_Y_*. In both cases the significance of not satisfying one of the above conditions is that increasing *m*, in the case of CPI, or *n*, in the case of CS, has detrimental effects on one of the specificity indicators, or increases one of the cross-terms *X_out_*|*Y_in _*or *Y_out_*|*X_in_*. The consequence of equation (7.2) is that networks with either CPI or CS can only utilize ultrasensitivity to decrease one of the crosstalk terms, *X_out_*|*Y_in _*or *Y_out_*|*X_in_*, at a time where the other cross-term must be kept small via cross-regulation. These beneficial effects of ultrasensitivity, however, greatly exceed those in linear or hyperbolic pathways or even those due to cross-regulation. The cross-terms that ultrasensitivity is able to decrease show polynomial decrease (and hence polynomial increase in corresponding specificity indicators) whereas the cross-terms that cross-regulation decrease, in the cases of CS and CPI, show only linear decrease (leading to a linear increase in corresponding specificity indicators). Thus due to the fact that *MFMS *is a minimum of the four specificity indicators (1.1), the bounds for CPI and CS show only linear increase with *CRT*.

In marked contrast to the above, scaffolding/compartmentalization allows for all four equations to be simultaneously satisfied. SC creates two distinct species of the shared component and therefore the derivatives with respect to Hill Exponents change to:

(7.3) $$\begin{array}{cc} \frac{\partial S_{X}}{\partial n}>0\Leftrightarrow x_{1}^{N}|X_{in}>\varepsilon_{X} & \frac{\partial S_{Y}}{\partial n}>0\Leftrightarrow x_{1}^{N}|Y_{in}<\varepsilon_{X}\\ \frac{\partial S_{X}}{\partial m}>0\Leftrightarrow x_{1}^{C}|X_{in}<\varepsilon_{Y} & \frac{\partial S_{Y}}{\partial m}>0\Leftrightarrow x_{1}^{C}|Y_{in}>\varepsilon_{Y} \end{array}$$

Combining these new equations gives:

(7.4) $$\begin{array}{ll} x_{1}^{N}|Y_{in}<\varepsilon_{X}<x_{1}^{N}|X_{in},\; & x_{1}^{C}|X_{in}<\varepsilon_{Y}<x_{1}^{C}|Y_{in} \end{array}.$$

This allows for the possibility of both *n *and *m *to increase *MFMS*. Therefore with scaffolding/compartmentalization, ultrasensitivity in both the *X *and *Y *pathways can simultaneously increase specificity.

Why does SC do so much better? Recall the issue in achieving mutual fidelity: if *X_out _*and *Y_out _*are activated in a monotone way mutual fidelity is impossible. Embellishing the system with insulation mechanisms is a way around this problem and hence insulation mechanisms are responsible for achieving *MFMS*. However in every case but SC, the problem remains that one of the *x*_1 _steady states must be lower than the other. For this reason there is no way to set the parameters so that increasing the ultrasensitivity simultaneously increases pathway specific variables, *X*_*out*_|*X*_*in *_and *Y_out_*|*Y_in_*, while decreasing the crosstalk terms, *X_out_*|*Y_in _*and *Y_out_*|*X_in_*. In SC, because the X pathway is only activated by $x_{1}^{N}$ and the Y pathway is only activated by $x_{1}^{C}$ one can set the threshold for $x_{1}^{N}$ input such that it is above the steady state level when given Y input but below the steady state level when given X input, and visa versa for the $x_{1}^{C}$ threshold. The consequences of this are that in this case ultrasensitivity can simultaneously decrease both cross-terms which allows for a polynomial decrease in both terms and thus a polynomial increase in *MFMS *as a whole, as seen in Figure 3C and equation (6.4). For example for *n *= 2 SC achieves *MFMS *at a level an order of magnitude higher than either CPI or CS.

### 8. Normalization

Specificity in cell signaling pathways is often easy to observe. For instance, yeast cells mate when exposed to mating pheromone and form filaments when starved for nutrients. However this is an observation of whole cell behavior that either happens or not. Quantifying specificity is a more difficult task. Typically one measures the level of pathway specific outputs.

Specificity is defined here as a ratio of two different variables, *X *and *Y*, which represent the output of the *X *and *Y *pathways, respectively. When measuring this output from a real cell a common thing to measure would be a concentration of an activated kinase or the transcript of a pathway-specific gene; let us call this concentration of gene *X*. However when comparing this to the output from another pathway, one would be dividing concentration of gene *X *by the concentration of gene (or kinase) *Y*. But the concentration at which gene *X *triggers a physiological endpoint, like mating, may be very different, potentially by orders of magnitude, than the concentration at which gene *Y *triggers a different output.

To address this issue the two output variables must be normalized somehow so that the construct *X_out_*|*X_in _*is not given in units of concentration of a pathway-specific gene, but given in a unit-less percent of a *characteristic *concentration for this gene. Two reasonable choices for a characteristic concentration would be the basal level of activation of the gene under no input, or the maximal or steady state level of activation of the gene under its own input. Each choice of a characteristic level should be specific to the system being studied, and hence in the analysis above we assume that such a choice has already been made and the variables are fittingly normalized.

The choice of normalization can have mathematical consequences that lead to a reinterpretation of data. For this reason we discuss the consequences of normalizing by the steady state levels of activation (for a discussion of normalization using basal levels see [25]).

To do this we use as a characteristic value the steady state value of *X_out_*|*X_in _*for the X pathway and *Y_out_*|*Y_in _*for the Y pathway and we define new normalized variables, denoted ${\overset{\wedge }{X}}_{out}|X_{in}$:

$$\begin{array}{ll} {\overset{\wedge }{X}}_{out}|X_{in}=\frac{X_{out}|X_{in}}{X_{out}|X_{in}}=1 & {\overset{\wedge }{X}}_{out}|Y_{in}=\frac{X_{out}|Y_{in}}{X_{out}|X_{in}}\\ {\overset{\wedge }{Y}}_{out}|X_{in}=\frac{Y_{out}|X_{in}}{Y_{out}|Y_{in}} & {\overset{\wedge }{Y}}_{out}|Y_{in}=\frac{Y_{out}|Y_{in}}{Y_{out}|Y_{in}}=1 \end{array}.$$

With these new definitions of normalized variables we calculate the output specificity

$${\overset{\wedge }{S}}_{X}=\frac{{\overset{\wedge }{X}}_{out}|X_{in}}{{\overset{\wedge }{Y}}_{out}|X_{in}}=\frac{X_{out}|X_{in}}{X_{out}|X_{in}}\cdot \frac{Y_{out}|Y_{in}}{Y_{out}|X_{in}}=\frac{Y_{out}|Y_{in}}{Y_{out}|X_{in}}={\overset{\wedge }{F}}_{Y}$$

and

$${\overset{\wedge }{S}}_{Y}=\frac{{\overset{\wedge }{Y}}_{out}|Y_{in}}{{\overset{\wedge }{X}}_{out}|Y_{in}}=\frac{Y_{out}|Y_{in}}{Y_{out}|Y_{in}}\cdot \frac{X_{out}|X_{in}}{X_{out}|Y_{in}}=\frac{X_{out}|X_{in}}{X_{out}|Y_{in}}={\overset{\wedge }{F}}_{X}.$$

So in this case input fidelity in the X pathway is the same as output specificity in the Y pathway and visa versa. Further the idea of mutual specificity and mutual fidelity are one in the same.

How does this reduction effect the conclusions above? First, clearly mutual specificity is no longer possible without cross regulation, since mutual specificity and fidelity are now equated. The reason that this was possible before and is no longer possible is that attaining *MS *without cross regulation requires the maximal output of one of the pathways to become large, however with the new normalized species this is impossible as they are both bounded by one.

Secondly the bounds that we derived on MFMS, or more simply specificity in this case, still hold. In each case the degree of MFMS was limited by fidelity, see above. Recall that with no cross regulation mutual specificity of any degree is possible (see section 3) and in fact in this case it is possible to simultaneously maximize *S_X_*, *S_Y _*and *F_X _*but not *F_Y _*(see section 1a of the Additional file 1). Hence the reduction of the problem of achieving *MFMS *to achieving *MF *does not make the problem easier.

With a normalization such as this we get a great reduction in the equations to consider. Further the normalized construct makes some intuitive sense. The outputs are given in terms of percent of the activation that occurs when the appropriate input is given. So why not always express the outputs this way? The terms used to normalize the outputs, *X_out_*|*Y_in _*and *Y_out_*|*Y_in_*, are dependent on the input strength. So for a small input the variables are normalized to a smaller number (making them larger) whereas if a large input is used it is the opposite case. Also the input strength of each pathway can be independently varied. So it is possible to choose a strong input for the × pathway and a weak input for the Y pathway or visa versa. These choices could then alter the values of specificity in the system. Again the units of the input to each system is potentially different and hence comparing them is inappropriate. So we are again faced with the problem of normalizing the input based on some characteristic value.

The simple explanation to these issues is that the problem of normalization can be complicated and should be considered on a case-by-case basis. Here we assume that this has been done and all of the variables are unit-less.

## Conclusion

Cell signaling is integral to numerous fundamental biological processes including development, mating, multicellularity, learning and memory, and many others. In addition, defects in cell-cell communication and signal transduction are central to the pathogenesis of many human diseases, such as cancer and diabetes. A quantitative understanding of the properties of cell signaling is of critical importance both for greater basic understanding and for the development of new clinical paradigms. A major obstacle to this goal, however, is the challenge of understanding the design logic underlying the complicated, interconnected networks in which most signaling pathways are embedded.

Here we have focused on mechanisms that provide specificity to interconnected networks containing distinct pathways that share components. We combined a framework for the analysis of specificity in cell signaling with simple mathematical models of interconnected networks. Using this approach, we examined how the stimulus-response properties of signaling pathways may influence their specificity. We compared weakly-activated pathways with hyperbolic or ultrasensitive pathways, asking if they could provide or enhance specificity, either alone or when combined with certain insulating mechanisms. We found that ultrasensitivity could not provide specificity on its own, but could enhance the performance of certain insulating mechanisms.

### Ultrasensitivity can confer output specificity but not input fidelity

To measure specificity we focused on two metrics, *mutual output specificity *(*MS*), where both pathways preferentially activate their own outputs, and *mutual input fidelity *(*MF*), where both pathways preferentially respond to their own inputs. Examining the network denoted the "basic architecture", a generic, idealized network containing two pathways that share a component, we found that mutual fidelity is impossible at steady-state. Previously we showed that weakly-activated pathways can endow this architecture with neither mutual specificity nor mutual fidelity [22,25]. Here, we significantly extended this finding by showing that, while both hyperbolic and ultrasensitive pathways can provide mutual specificity, neither can provide mutual fidelity. In fact, our analysis applies not only to hyperbolic and ultrasensitive pathways, but also to any monotonic stimulus-response (input-output) relationship, for reasons discussed below.

Why is input fidelity more difficult to obtain than output specificity? Consider the input-output functions for the *X *and *Y *pathways. When both input-output functions are straight lines (as they are when both pathways are weakly activated), they cannot cross; that is, they intersect at the origin but nowhere else. Therefore, it is impossible to choose a pair of input levels so that *X *output is greater than *Y *at the lower level of input, and *Y *output is greater than *X *at the higher level of input (or visa versa). In other words, mutual specificity is impossible. The input-output functions corresponding to hyperbolic and ultrasensitive pathways are curves, not straight lines. Hence, one can pick parameters such that these curves will cross at some point, and thence it will be possible to pick a pair of input levels on either side of the intersection point that will provide mutual specificity. Mutual fidelity, in contrast, can only be obtained if one of the input-output curves reaches a maximum and then bends back down (see Fig. 2); that is, this curve must be non-monotonic. Non-monotonic behavior in stimulus-response curves generally cannot be achieved by cascades of enzymes that exhibit standard kinetics (even if there is cooperativity or multisite phosphorylation); instead, some sort of negative feedback loop or cross-inhibition will be needed [51]. In this sense, then, mutual input fidelity is more difficult to achieve, and also, perhaps, more difficult to evolve.

Another implication of this result is that, for interconnected networks to exhibit input fidelity (wherein pathways respond preferentially to authentic inputs), the basic network architecture must be embellished with an insulating mechanism(s). This insulating mechanism may work, for instance, by transforming one the stimulus-response curves into a non-monotonic function (e.g. cross-pathway inhibition), or it may act by splitting the stimulus (e.g. scaffolding/compartmentalization) or by splitting a single response curve into two (e.g. combinatorial signaling). Regardless, we should expect insulating mechanisms to be found wherever pathways share components yet exhibit specificity from signal to cellular response.

### Ultrasensitivity dramatically improves the performance of insulating mechanisms

Although ultrasensitivity cannot provide mutual fidelity to the basic architecture by itself, it can significantly improve the performance of several different insulating mechanisms. To facilitate comparison, we defined a term denoted the Cross Regulatory Term (CRT) for each of the different insulating mechanisms. In every case this was a non-dimensional term that characterized the "strength" of the insulating mechanism. This allowed us to quantify the degree to which mutual specificity and mutual fidelity (*MFMS*) increased as a result of increases in the CRT. We found that, as the CRT was increased, there was a much sharper increase in *MFMS *in those networks featuring ultrasensitive activation than in those with linear (i.e. weak) or hyperbolic activation.

All the networks that we examined displayed sharper increases in *MFMS *with ultrasensitive activation than with linear or hyperbolic activation. However, for the networks that utilized combinatorial signaling or cross-pathway inhibition, it was not possible to utilize ultrasensitivity simultaneously in both pathways to the benefit of specificity. In other words, increasing the ultrasensitivity in one of the pathways was detrimental. In contrast, networks that combined scaffolding/compartmentalization with ultrasensitive activation could achieve very high levels of *MFMS *even at low levels of cross regulation, and ultrasensitivity in both pathways was beneficial.

To summarize, the hierarchy we have found is as follows: First, in the absence of an insulating mechanism and in the presence of linear activation, neither mutual specificity nor mutual fidelity is possible. Second, the addition of ultrasensitive activation allows for mutual specificity; in fact, with a careful selection of parameters, *S*_*X*_, *S*_*Y *_and *F*_*X *_can become unbounded. However, mutual fidelity still cannot be achieved. Third, the addition of cross-pathway inhibition or combinatorial signaling to an ultrasensitive system can achieve mutual fidelity, but with a linear dependence on the amount of cross-regulation. Finally, ultrasensitive systems utilizing scaffolding/compartmentalization can realize a super-linear increase in mutual specificity and mutual fidelity as the extent of cross-regulation is increased.

### Constraints and opportunities

During evolution, as new signaling pathways emerged from the duplication and divergence of pre-exiting parts, the issue of specificity must have been paramount. Why component sharing is a widespread feature of cellular regulatory networks is a mystery. One possibility is that some low level of crosstalk between pathways is beneficial, but too much is bad; this would explain the existence of both crosstalk and insulating mechanisms. Another possibility is that duplication of part of a pathway, followed by the imposition of an insulating mechanism, is an easier evolutionary path to take than duplication of an entire pathway. Regardless, it seems possible that some of the constraints (e.g. input fidelity is hard to achieve) and opportunities (e.g. ultrasensitivity can help the performance of insulating mechanisms) identified here may have influenced the evolution of signal transduction networks.

## Methods

See Additional file 1.

## Authors' contributions

SH carried out model development, simulation, analysis, and wrote the manuscript. LB and QN helped with model formulation and analysis and co-wrote the manuscript. All authors read and approved the final manuscript.

## Supplementary Material

Additional file 1 **Supplementary Materials - Ultrasensitive Reponses and Specificity in Cell Signaling**. Derivation for equations and supplementary figures. Click here for file
