# Supplementary material for: Ultrasensitive Responses and Specificity in Cell Signaling
Source: BMC Syst Biol. 2010 Aug 25;4:119. doi: 10.1186/1752-0509-4-119 (PMC2940771; doi:10.1186/1752-0509-4-119)
Supplement: Additional file 1 — Supplementary Materials - Ultrasensitive Reponses and Specificity in Cell Signaling. Derivation for equations and supplementary figures. [file 1752-0509-4-119-S1.PDF]

Ultrasensitive Responses and Specificity in Cell Signaling: Supplementary Material

Seth Haney[1], Lee Bardwell[2,3,4*] and Qing Nie[1,3,4*]

[1]Department of Mathematics

[2]Department of Developmental and Cell Biology

[3]Center for Complex Biological Systems

[4]Center for Mathematical and Computational Biology

University of California at Irvine, Irvine, CA 92697, USA

*Corresponding authors

Email addresses:

SH: shaney@uci.edu

LB: bardwell@uci.edu

QN: qnie@uci.edu

1. *Unbounded Mutual Specificity with Ultrasensitive Activation*

a. *Proof of that Unbounded MS is Possible in Hyperbolic case*

Here we show that it is possible to attain mutual specificity of any degree with ultrasensitive or hyperbolic activation and no cross regulation. To do this we create a sequence of parameters indexed by k for which MS will approach infinity when $k \to \infty$. When doing this we want to ensure two important properties we describe below.

First we ensure that the pathway specific variables do not vanish. In other words we do not want $X_{out} \mid X_{in} \xrightarrow{k\to\infty} 0$ or $Y_{out} \mid Y_{in} \xrightarrow{k\to\infty} 0$. The reason we stress this point is that specificity is meaningless if attaining it requires the intended output of the pathway to vanish, since this essentially would only achieve specificity by completely shutting down one pathway. To do this we set a threshold value, *T*, so that each of these variables stays above this threshold.

Secondly we want to ensure that the crosstalk terms $X_{out} \mid Y_{in}$ and $Y_{out} \mid X_{in}$ remain bounded. In other words we want it to be the case that $X_{out} \mid Y_{in} < M$ and $Y_{out} \mid X_{in} < M$, for some finite *M*. If this were not the case, it would imply that specificity is attained by simply making the system extremely sensitive to input and while the pathway specific variables become large so too do the crosstalk terms.

So the specificity indicators in this case are:

$$S_X = \frac{\alpha}{\beta}\frac{1+(\varepsilon_Y / x_1^X)^m}{1+(\varepsilon_X / x_1^X)^n} \quad S_Y = \frac{\beta}{\alpha}\frac{1+(\varepsilon_X / x_1^Y)^n}{1+(\varepsilon_Y / x_1^Y)^m}.$$

We will do this for the case when $n = m = 1$ which will show that this is possible in both ultrasensitive and hyperbolic pathways. Let, $\alpha = x_1^X = \varepsilon_X = k$, $\beta = 2T$, and $x_1^Y = \varepsilon_Y = 1/k$. Then

$$
\begin{aligned}
S_X &= \langle X_{out} \mid X_{in} \rangle / \langle Y_{out} \mid Y_{in} \rangle \\
&= \frac{k}{1} \cdot \frac{1+1/k^2}{2T} \\
&= \frac{k+1/k}{2T} \\
&\to \infty \qquad\qquad . \\
S_Y &= \langle Y_{out} \mid Y_{in} \rangle / \langle X_{out} \mid Y_{in} \rangle \\
&= \frac{T}{1} \cdot \frac{1+k^2}{k} \\
&= T(1/k+k) \\
&\to \infty .
\end{aligned}
$$

Therefore mutual specificity of any degree can be obtained. Further note that the pathway specific variables, $X_{out} \mid X_{in}$ and $Y_{out} \mid Y_{in}$ do not vanish:

$$
\begin{aligned}
X_{out} \mid X_{in} &= \frac{\alpha}{1+\varepsilon_X / x_1^X} \\
&= \frac{k}{1+1} \\
&\to \infty \\
Y_{out} \mid Y_{in} &= \frac{\beta}{1+\varepsilon_Y / x_1^Y} \\
&= \frac{2T}{(1+1)} \\
&= T .
\end{aligned}
$$

Here $X_{out} \mid X_{in}$ grows to infinity and $Y_{out} \mid Y_{in}$ stays at a constant level, *T*. Further the crosstalk terms remain bounded:

$$
\begin{aligned}
X_{out} \mid Y_{in} &= \frac{\alpha}{1+\varepsilon_X / x_1^Y} \\
&= \frac{k}{1+k^2} \\
&\to 0 \\
Y_{out} \mid X_{in} &= \frac{\beta}{1+\varepsilon_Y / x_1^X} \\
&= \frac{2T}{1+1/k^2} \\
&\to 2T
\end{aligned}
$$

where here the $X_{out} \mid Y_{in}$ vanishes and the $Y_{out} \mid X_{in}$ term remains bounded by the constant *2T.*

b. *Discussion of Effects of Ultrasensitivity on Obtaining Specificity when Bounds Remain the Same*

Unbounded MS is obtainable in either the ultrasensitive or hyperbolic case. So what advantage do pathways with ultrasensitivity have over hyperbolic pathways? As is the case with bounds on $MFMS$, ultrasensitivity increases the speed at which networks can approach these bounds. What this means is that for a given degree of MS or $MFMS$ less severe restrictions on the parameters are required to obtain this degree in the case where ultrasensitivity is present. Why is this important? To obtain levels of MS or $MFMS$ closer to the bounds requires some parameters to approach zero or infinity. In a cell signaling system, these parameters represent real physical quantities such as decay and production rates that which will have upper and lower limits due to energy and other constraints. So in all likelihood many of these parameters will not be able to approach zero or infinity. Further the physical quantities that the parameters represent, activation rates etc., have effects on other aspects of a functioning cell and so extreme values may hinder other cellular processes. Therefore a cell signaling system with ultrasensitive activation, which allows for less stringent restraints on parameters to obtain high levels of specificity has a significant advantage over hyperbolic pathways.

To formalize this idea in a more quantitative sense: ultrasensitive pathways can show a polynomial decrease of cross-terms $X_{out} \mid Y_{in}$ or $Y_{out} \mid X_{in}$, provided the parameters are set appropriately, where as cross-terms in hyperbolic pathways can at best show a linear decrease. Further the cross-terms not decreased by ultrasensitivity ultrasensitive pathways, where cross-regulation is needed, also show only a linear decrease (for example see the $Y_{out} \mid X_{in}$ term in CPI or the $X_{out} \mid Y_{in}$ term in CS).

2. *Sharpness of Bounds on MFMS*

In this section we show that the bounds for $MFMS$ derived in section 6 of the main text are sharp. By sharp we mean that this is the least upper bound for $MFMS$, or more formally, for any level of $MFMS$ less than the bound there is a set of parameters that will achieve that level. We will do this for the case of CPI only, the case for CS is similar. As before we create a sequence of parameters indexed by k for which $MFMS$ will approach the bound, $1+CRT$, when $k \to \infty$. First recall the specificity indicators in the case of CPI:

$$S_X = \left(\frac{\alpha}{\beta}\right)\frac{f^X(x_1^X)}{f^Y(x_1^X)}[1+CRT\cdot f^X(x_1^X)] \quad S_Y = \left(\frac{\beta}{\alpha}\right)\frac{f^Y(x_1^Y)}{f^X(x_1^Y)}\frac{1}{1+CRT\cdot f^X(x_1^Y)}$$
$$F_X = \frac{f^X(x_1^X)}{f^X(x_1^Y)} \qquad F_Y = \frac{f^Y(x_1^Y)}{f^Y(x_1^X)}\frac{1+CRT\cdot f^X(x_1^X)}{1+CRT\cdot f^X(x_1^Y)}.$$

We will show that the bound holds even for the hyperbolic case, $n=m=1$, the case of arbitrary *n* and *m* can be done similarly.

Let $\varepsilon_Y = k$, $x_1^Y = k^2$, $\varepsilon_X = k^3$, $x_1^X = k^4$ and $\alpha = \beta$. Then

$$
\begin{aligned}
S_X &= \frac{1+(\varepsilon_Y / x_1^X)^m}{1+(\varepsilon_X / x_1^X)^n}\left[1+CRT\frac{1}{1+(\varepsilon_X / x_1^X)^n}\right] \\
&= \frac{1+k^{-3}}{1+k^{-1}}\left[1+CRT\frac{1}{1+k^{-1}}\right]\xrightarrow{k\to\infty} 1\cdot\left[1+CRT\cdot 1\right] \\
&= 1+CRT
\end{aligned}
$$

$$
\begin{aligned}
S_Y &= \frac{1+(\varepsilon_X / x_1^Y)^n}{1+(\varepsilon_Y / x_1^Y)^m}\left[\frac{1}{1+CRT\frac{1}{1+(\varepsilon_X / x_1^Y)^n}}\right] \\
&= \frac{1+k}{1+k^{-1}}\frac{1}{1+CRT\frac{1}{1+k^{-1}}}\xrightarrow{k\to\infty}\infty\cdot 1 \\
&= \infty
\end{aligned}
$$

$$
\begin{aligned}
F_X &= \frac{1+(\varepsilon_X / x_1^Y)^n}{1+(\varepsilon_X / x_1^X)^n} \\
&= \frac{1+k}{1+k^{-1}}\xrightarrow{k\to\infty}\infty
\end{aligned}
$$

$$
\begin{aligned}
F_Y &= \frac{1+(\varepsilon_Y / x_1^X)^m}{1+(\varepsilon_Y / x_1^Y)^m}\left[\frac{1+CRT\frac{1}{1+(\varepsilon_X / x_1^X)^n}}{1+CRT\frac{1}{1+(\varepsilon_X / x_1^Y)^n}}\right] \\
&= \frac{1+k^{-3}}{1+k^{-1}}\left[\frac{1+CRT\frac{1}{1+k^{-1}}}{1+CRT\frac{1}{1+k}}\right] \\
&\xrightarrow{k\to\infty} 1+CRT.
\end{aligned}
$$

Thus taking the minimum over these four quantities we get $MFMS \to 1+CRT$. Therefore the bound is sharp. A similar argument will show this is the case for CS as well.

3. *Bi-Directional Insulation Mechanisms*

Here we examine networks where IM are imposed in both directions. Specifically we will analyze a) bi-directional CS: where CS is applied to both pathways, b) simultaneous CS and CPI where CPI acts from the X pathway to the Y pathway and CS acts on the X pathway, and c) bi-directional CPI: when CPI is applied both from the X pathway to the Y pathway as well as from the Y pathway to the X pathway. We do this by simultaneously modifying both the x_2 and y_2 equations. For example for simultaneous CS and CPI the equations are

$$\begin{aligned} \dot{x}_2 &= a_2 R[x_0] f^X(x_1) - d_2^x x_2 \\ \dot{y}_2 &= b_2 f^Y(x_1) \frac{1}{1 + x_2 / \varepsilon_g} - d_2^y y_2 \end{aligned}.$$

We examine the benefits over single mechanisms as well as the potential benefits ultrasensitivity may have. In each case we will denote the CRT in the X pathway, term quantifying the degree of cross-regulation applied *to* the X pathway, CRT_X and the corresponding term to the Y pathway CRT_Y, regardless of the type of cross-regulation (recall section 5 from the main text, that the CRTs are dimensionless terms thus comparable).

a. *CS-CS*

For bi-directional CS it is easy to calculate the specificity indicators:

$$\begin{aligned} S_X &= \left(\frac{\alpha}{\beta}\right) \frac{f^X(x_1^X)}{f^Y(x_1^X)} CRT_Y \quad & S_Y &= \left(\frac{\beta}{\alpha}\right) \frac{f^Y(x_1^Y)}{f^X(x_1^Y)} CRT_X \\ F_X &= \frac{f^X(x_1^X)}{f^X(x_1^Y)} CRT_X & F_Y &= \frac{f^Y(x_1^Y)}{f^Y(x_1^X)} CRT_Y \end{aligned}.$$

Now either $x_1^X > x_1^Y$ or $x_1^Y > x_1^X$. In the first case, since f^X and f^Y are monotonic, $f^Y(x_1^Y) < f^Y(x_1^X)$ and thus

$$F_Y = \frac{f^Y(x_1^Y)}{f^Y(x_1^X)} CRT_Y < CRT_Y .$$

Alternatively $x_1^Y > x_1^X$ and $f^X(x_1^X) < f^X(x_1^Y)$ implying

$$F_X = \frac{f^X(x_1^X)}{f^X(x_1^Y)} CRT_X < CRT_X .$$

In either case $MFMS < \max\{CRT_X, CRT_Y\}$ or in the case that these two levels are equal

$$MFMS < CRT .$$

This is the *same* bound we calculated for one-way Combinatorial Signaling, and likewise it is sharp or the least upper bound. Further when the derivative analysis is performed, as in Section 8 in the main text, we arrive at the same result as in the main text. For specificity to be increased by both m and n it must be that

$$x_1^X < \varepsilon_Y < x_1^Y < \varepsilon_X < x_1^X$$

which as before is impossible.

b. *CPI-CS.*

In this case we investigate a network with CPI which acts from the X pathway to the Y pathway and CS which acts on the X pathway. We calculate the specificity indicators,

$$S_X = \left(\frac{\alpha}{\beta}\right)\frac{f^X(x_1^X)}{f^Y(x_1^X)}[1 + CRT_Y f^X(x_1^X)] \quad S_Y = \left(\frac{\beta}{\alpha}\right)\frac{f^Y(x_1^Y)}{f^X(x_1^Y)}\frac{CRT_X}{1 + CRT_Y f^X(x_1^Y)}$$

$$F_X = \frac{f^X(x_1^X)}{f^X(x_1^Y)} CRT_X \qquad F_Y = \frac{f^Y(x_1^Y)}{f^Y(x_1^X)}\frac{1 + CRT_Y f^X(x_1^X)}{1 + CRT_Y f^X(x_1^Y)}$$

Using similar logic to the bi-directional CS case above, we find

$$MFMS < \max\{CRT_X, 1 + CRT_Y\},$$

which is just the maximum of the two bounds for the one directional CS and CPI, respectively. Also the derivative analysis, as in Section 8 in the main text, gives the same

result, in that the combination of CPI with CS does not solve the problem described in the main text.

To examine networks with bi-directional CPI the analysis becomes difficult. While steady state solutions do exist, they are cumbersome and it is easier to work with numerical solutions, see next section.

c. Numerical Solutions and Conclusions.

From the analysis above we have shown that bi-directional insulation mechanisms will not aid in raising the maximal $MFMS$ value in terms of the maximal, X or Y pathway, CRT. Further ultrasensitivity will only help in achieving higher $MFMS$ if the parameters are chosen appropriately, as in section 8 in the main text.

To better understand this we numerically evaluated the specificity indicators for each of the networks above over a large range of parameters, see Figure S1. The CS-CS and CPI-CS networks were done using steady state values calculated analytically and the CPI-CPI networks were done by approximating the steady state values using numerical solutions to the ordinary differential equations.

It is obvious from Figure S1 that the only element that determines whether ultrasensitivity in the X or Y pathway will be beneficial for increasing $MFMS$ is whether the parameters were chosen so that $\varepsilon_Y < x_1^Y < \varepsilon_X < x_1^X$ or $\varepsilon_X < x_1^X < \varepsilon_Y < x_1^Y$, abbreviated x1x>x1y and x1y>x1x in the figures. As before, if the parameters are in the opposing orientation for a particular pathway then raising the ultrasensitivity actually detracts from $MFMS$.

When comparing bi-directional insulation mechanisms to their one directional counterparts we see again that parameters are the main factor in determining whether ultrasensitivity increases or decreases specificity. Also, somewhat surprisingly, bi-directional insulation mechanisms do not provide a significant increase in maximal

$MFMS$ over one directional insulation mechanisms, Figure S2. This is consistent with the analytical results.

It is important to realize that this does not imply that bi-directional insulation mechanisms give no advantage over one directional mechanisms, in fact the addition only increases the specificity indicators. However the maximal specificity obtainable is the same. So if the parameters are not optimized to obtain the highest degree of specificity bi-directional insulation mechanisms will increase $MFMS$. For example in the case of bi-directional CS it is clear that S_X and F_Y are increased by the addition of combinatorial signaling in the Y pathway, i.e. an increase over combinatorial signaling as defined in the main text, but since these two specificity indicators are not the limiting components in $MFMS$ when parameters are optimized the maximal bounds are no different.

4. Derivatives with Respect to Hill Exponents

a. Standard Model

To derive the conditions to determine when ultra-sensitivity can increase specificity, we simply took derivatives of the steady states of S_X and S_Y. Similarly one can take the derivatives of the steady states ($\langle X \mid X \rangle$, etc.) to derive the same results.

$$\frac{\partial S_X}{\partial n} = \frac{\partial}{\partial n}\left(\frac{X \mid X}{Y \mid X}\right) = \frac{1}{Y \mid X}\frac{\partial}{\partial n}\left(\alpha \frac{x_1^n}{\varepsilon_x^n + x_1^n}\right) = \frac{\alpha}{Y \mid X}\frac{\partial}{\partial n}\left(\frac{1}{\left(\varepsilon_x / x_1\right)^n + 1}\right)$$

$$= \frac{-\alpha}{Y \mid X}\frac{\ln\left(\varepsilon_x / x_1\right)}{\left(\left(\varepsilon_x / x\right)^n + 1\right)} = \gamma \ln\left(x_1 / \varepsilon_x\right)$$

where γ is a positive term. Hence the sign of the derivative is completely determined by the sign of $\ln(x_1 / \varepsilon_x)$, thus is positive if and only if $x_1 > \varepsilon_x$. The remaining conditions are derived analogously.

In the case of CS and CPI the conditions on the sign of these derivatives are exactly the same as in the standard model, the magnitudes of the derivatives do change however.

Figures

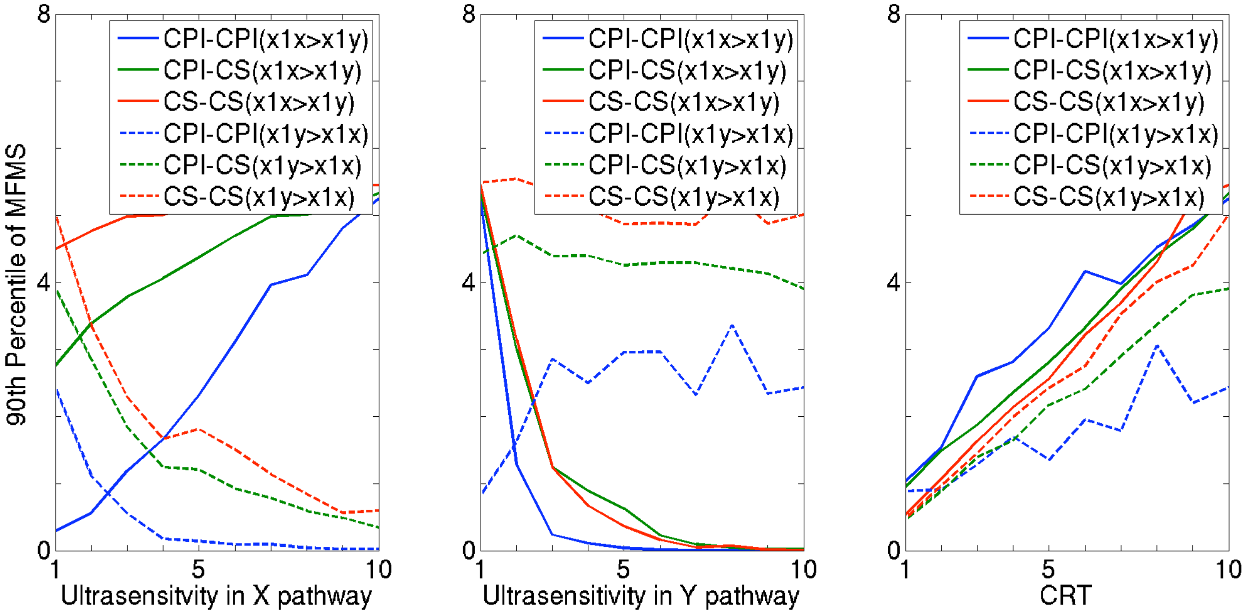

Figure S1 - Bi-directional Insulation Mechanisms

MFMS values for networks with CS-CS, CPI-CS, and bi-CPI were calculated for 10,000 trials over a large range of parameters. *MFMS* values at the 90th percentile of the distribution were then plotted as a function of degree of various Hill exponents. **(Left)** Dependence of each type of network on *n*, the degree of ultrasensitivity in the *X* pathway. **(Middle)** Dependence on *m*, the ultrasensitivity of the Y pathway. **(Right)** Dependence on CRT, where $CRT \equiv CRT_X = CRT_Y$.

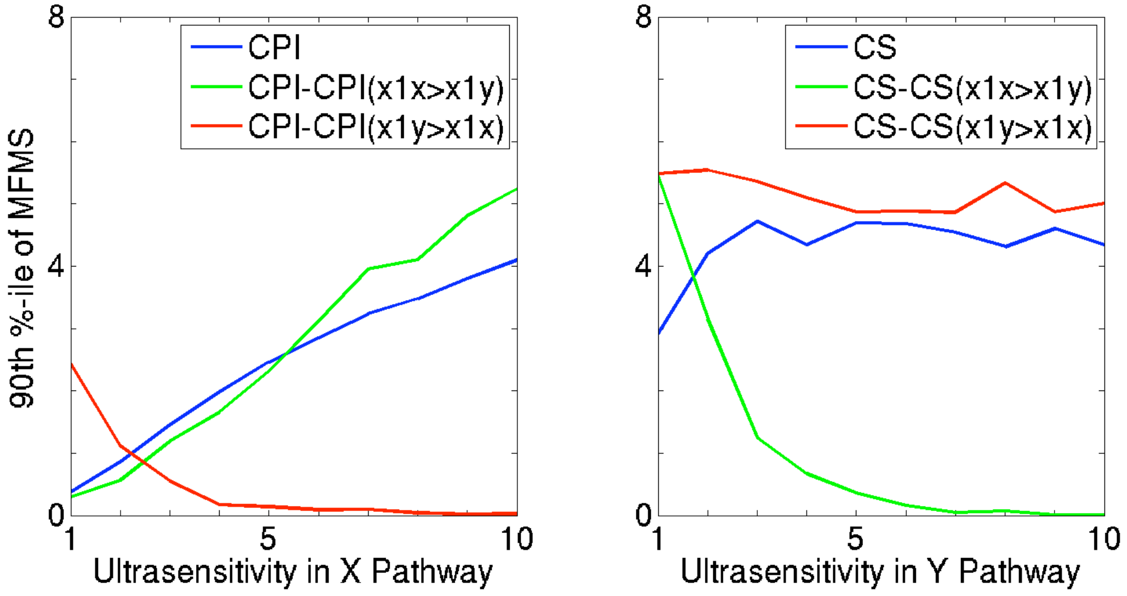

Figure S2 - Comparison with One-directional Insulation Mechanisms.

MFMS values at the 90th percentile of the distribution were then plotted as a function of degree of various Hill exponents. **(Left)** Dependence of each type of network on *n*, the degree of ultrasensitivity in the *X* pathway for networks with CPI or CPI-CPI. **(Right)** Dependence on *m*, the ultrasensitivity of the Y pathway for networks with CS or CS-CS.
